# Improvement of image quality of diffusion-weighted imaging (DWI) with deep learning reconstruction of the pancreas: comparison with respiratory-gated conventional DWI

**DOI:** 10.1007/s11604-025-01790-w

**Published:** 2025-04-26

**Authors:** Kazuki Oyama, Fumihito Ichinohe, Yasuo Adachi, Yoshihiro Kito, Katsuya Maruyama, Minoru Mitsuda, Thomas Benkert, Omar Darwish, Yasunari Fujinaga

**Affiliations:** 1 Department of Radiology, Shinshu University School of Medicine, 3-1-1 Asahi, Matsumoto, Nagano 390-8621 Japan; 2 Radiology Division, Shinshu University Hospital, 3-1-1 Asahi, Matsumoto, Nagano 390-8621 Japan; 3 MR Research and Collaboration Department, Siemens Healthcare K.K, Tokyo, Japan; 4 Magnetic Resonance Department, Siemens Healthcare K.K, Tokyo, Japan; 5 MR Application Predevelopment, Siemens Healthcare AG, Forchheim, Germany

**Keywords:** Magnetic resonance imaging, Diffusion-weighted imaging, Deep learning-based reconstruction, Pancreas, Solid lesions

## Abstract

**Purpose:**

This study aimed to evaluate the efficacy of deep learning-based reconstruction (DLR) in improving pancreatic diffusion-weighted imaging (DWI) quality.

**Materials and methods:**

In total, 117 patients (mean age of 68.0 ± 12.9 years) suspected of pancreatic diseases underwent magnetic resonance imaging (MRI) between July and December 2023. MRI sequences included respiratory-gated conventional diffusion-weighted images (RGC-DWIs), respiratory-gated diffusion-weighted images with deep learning-based reconstruction (DLR) (RGDLR-DWIs), and breath-hold diffusion-weighted images with DLR (BHDLR-DWIs) (short TE and long TE equal to other DWIs) at a 3 T MR system. Among these patients, 27 had solid lesions. Two radiologists qualitatively assessed pancreatic shape, main pancreatic duct (MPD) visualization, and solid lesion conspicuity using a 5-point scale. Quantitative analysis included apparent diffusion coefficient (ADC) values for pancreatic parenchyma and solid lesions, signal-to-noise ratio (SNR), pancreas-to-muscle signal-intensity ratio (PM-SIR) and lesion-to-pancreas signal-intensity ratio (LP-SIR). Differences among DWI sequences were analyzed using Friedman’s and Bonferroni’s tests.

**Results:**

Qualitatively, BHDLR-DWIs (short TE) had the highest scores for pancreatic shape and MPD but lowest for solid lesions visibility, whereas RGDLR-DWIs had the highest score for solid lesions. Quantitatively, BHDLR-DWIs (short TE) had the lowest ADC values for pancreatic parenchyma and solid lesions, with the highest PM-SIR. There was no significant difference between BHDLR-DWIs (short TE) and RGDLR-DWIs for solid lesion ADC values. RGC-DWIs had the highest SNR, though differences from RGDLR-DWIs and BHDLR-DWIs (short TE) were not significant. Although LP-SIR in RGDLR-DWIs were the lowest, the difference was not significant.

**Conclusion:**

BHDLR-DWIs (short TE) provided the best pancreatic morphology image quality, whereas RGDLR-DWIs were superior for solid lesion detection.

## Introduction

Diffusion-weighted imaging (DWI) is a non-invasive, quantitative imaging technique used to measure tissue diffusion properties [1]. Pancreatic DWI is commonly used in clinical practice and plays an important role in diagnosing pancreatic diseases, including detecting malignant tumors and distinguishing between benign and malignant lesions [2–7]. However, DWI has several limitations, such as reduced visibility of background anatomic structures, unclear positional information, prolonged acquisition time, and susceptibility to motion and distortion artifacts [8, 9]. There are two methods of data acquisition in DWI, breath-holding (BH) and non-BH (such as respiratory-triggered or free-breathing), each with its distinct characteristics [10]. BH offers shorter acquisition times due to breath-holding time limits, and reduced motion artifacts, but signal-to-noise ratio (SNR) is lower compared to non-BH techniques. Conversely, non-BH offers higher SNR and thinner slices if longer acquisition time is allowed.

Recently, deep learning-based reconstruction (DLR) has been successfully applied to various abdominal organs, eliminating noise and enabling high-resolution, faster image reconstruction [11–16]. Despite these advancements, DLR has not been extensively explored in pancreatic imaging, particularly for detecting solid pancreatic lesions.

Therefore, the aim of this study was to evaluate the effectiveness of DLR in enhancing pancreatic DWI image quality by comparing DWI under different respiration and DLR conditions.

## Materials and methods

### Patients

**Fig. 1:**
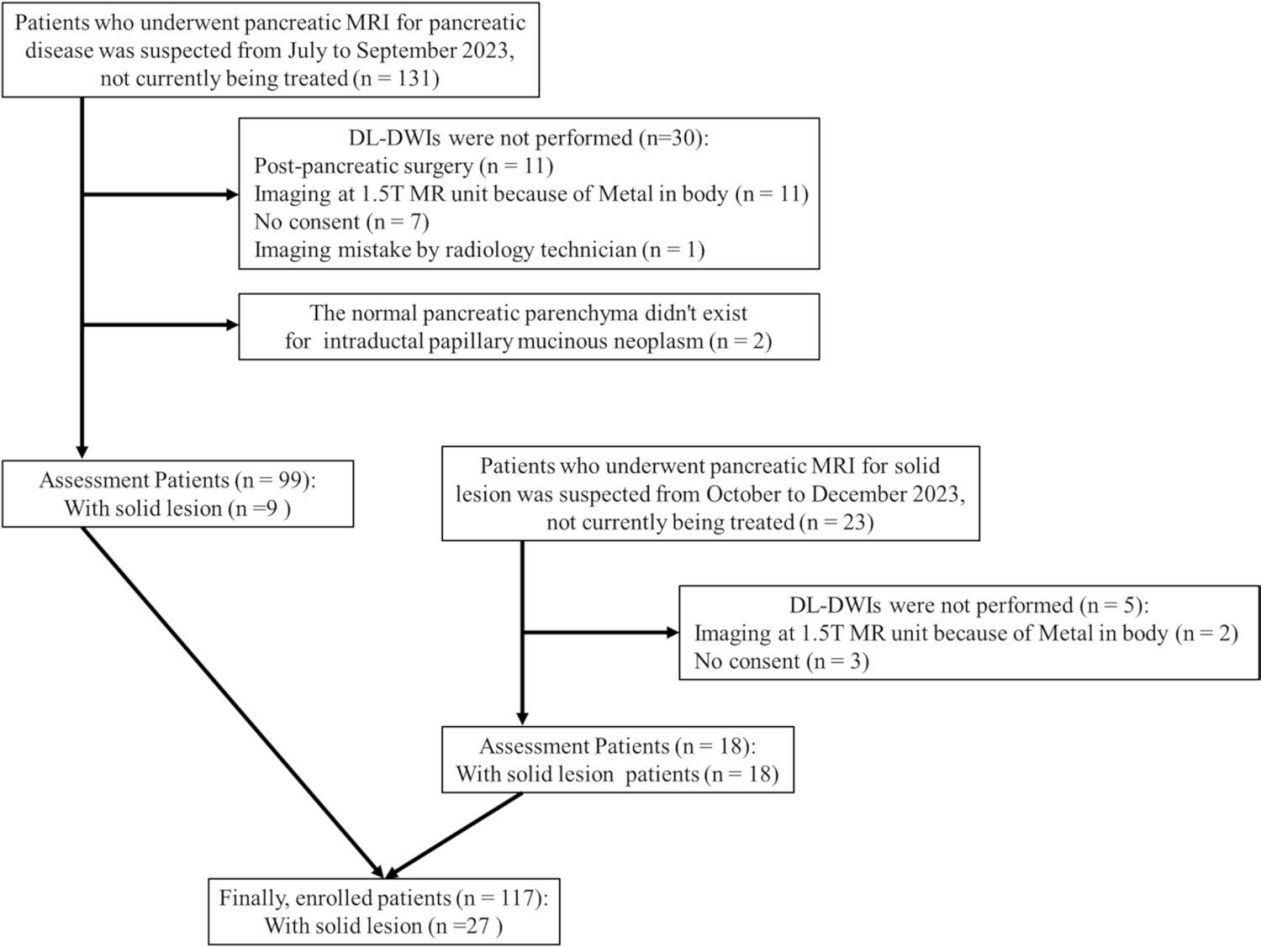
Summary flowchart illustrating the patient selection process

This prospective study was approved by our institutional review board. Written informed consent was obtained from all participants, and the procedures adhered to the ethical standards of the 1964 Declaration of Helsinki and its later amendments. The study involved two groups of patients. The first group included 131 consecutive patients suspected of pancreatic disease who underwent 3 T magnetic resonance imaging (MRI) not currently being treated between July and September 2023. Of these, 30 patients were excluded due to post-pancreatic surgery, presence of metal in the body, lack of consent, or imaging errors by the radiology technician. In addition, cases without normal pancreatic parenchyma due to intraductal papillary mucinous neoplasm (IPMN) were excluded, resulting in 99 patients with nine solid lesions. The second group included 23 consecutive patients suspected of having solid pancreatic lesions who underwent a 3 T MRI not currently being treated between October and December 2023. Five patients were excluded due to metal in the body or lack of consent, leaving 18 patients with 18 solid lesions. In total, 117 patients (39 males, 78 females; mean age ± standard deviation, 68.0 ± 12.9 years) with 27 solid lesions from both groups were evaluated. A flowchart of patient selection is shown in Fig. 1, and radiologic or pathological diagnoses are presented in Table 1.

**Table 1:** Clinical diagnosis

Clinical diagnosis	
Intraductal papillary mucinous neoplasm: without solid lesion (*n*)	53
with solid lesion (*n*)	4*
Other pancreatic cystic diseases (*n*)	15
Neuroendocrine neoplasm (*n*)	12*
Invasive ductal carcinoma (*n*)	7*
No abnormalities (*n*)	7
Chronic pancreatitis (*n*)	6
Serous cystic neoplasm (*n*)	3
Stenosis or dilation of the main pancreatic duct (*n*)	4
Intrapancreatic accessory spleen (*n*)	3*
Invasive intraductal papillary mucinous carcinoma (*n*)	1*
Signal abnormality (*n*)	1

*Disease with solid lesion

### MRI acquisition

All MRI scans were performed using a 3 T scanner (MAGNETOM Prisma; Siemens Healthineers, Erlangen, Germany) with 30-channel body array coil. Imaging included respiratory-gated conventional diffusion-weighted images (RGC-DWIs), research respiratory-gated diffusion-weighted images with DLR (RGDLR-DWIs), and research BH diffusion-weighted images with DLR (BHDLR-DWIs). Four DWI sequences used single-shot echo-planar imaging. The imaging parameters are listed in Table 2. Four DWI sequences were obtained at b = 50 and 1000 s/mm2, with scan times of 3–5 min for RGC-DWIs, 2.5–4 min for RGDLR-DWIs, and 1 min for BHDLR-DWIs (two 20-s BHs). Diffusion mode 3-scan trace was used for RGC-DWIs and RGDLR-DWIs, and 3D-Diagonal for BHDLR-DWIs due to reduction of breath-holding time.

**Table 2:** Imaging parameters

	RGC-DWIs	RGDLR-DWIs	BHDLR-DWIs (short TE)	BHDLR-DWIs (long TE)
Repetition time (ms)/Echo time (ms)	3000–5000/65	3000–5000/65	2600/36	2600/65
Flip angle (degree)	90	90	90	90
Acquisition matrix size	140 × 96	140 × 96	140 × 102	140 × 102
Reconstruction matrix size	280 × 192	280 × 192	280 × 204	280 × 204
Slice thickness (mm)	4	4	4	4
Number of slices	36	36	36	36
Field of view (mm^2^)	380 × 325	380 × 325	380 × 347	380 × 347
b-values (s/mm^2^)	1000 (and 50)*	1000 (and 50)*	1000 (and 50)*	1000 (and 50)*
Number of averages	3 for b = 1000	2 for b = 1000	3 for b = 1000	3 for b = 1000
	1 for b = 50	1 for b = 50	1 for b = 50	1 for b = 50
Phase partial Fourier	7/8	7/8	7/8	7/8
Reconstruction mode	Conventional	Deep Learning	Deep Learning	Deep Learning
Parallel imaging factor	GRAPPA 2	GRAPPA 4	GRAPPA 4	GRAPPA 4
Scan time (min)	3–5	2.5–4	1 (20-s BH × 2)	1 (20-s BH × 2)
Respiratory status	Respiratory synchronization	Respiratory synchronization	BH	BH
Diffusion mode	3-scan trace	3-scan trace	3D-Diagonal	3D-Diagonal

*50 for ADC values calculation

*BH* breath-hold, *RGC-DWIs* respiratory-gated conventional diffusion-weighted images, *RGDLR-DWIs* research respiratory-gated diffusion-weighted images with deep learning reconstruction, *BHDLR-DWIs* research breath-hold diffusion-weighted images with deep learning reconstruction, *GRAPPA* GeneRalized Autocalibrating Partial Parallel Acquisition

### Deep learning-based k-space-to-image reconstruction

RGC-DWIs were reconstructed with conventional GRAPPA, whereas RGDLR-DWIs and BHDLR-DWIs were reconstructed using a deep learning-based research application approach. The processing involved two consecutive steps similar to existing literature [17, 18]: First, a deep learning reconstruction from k-space to image was performed, inspired by the variational network concept [19]. In this step, raw single-shot k-space data and precomputed coil sensitivity maps were processed through 17 unrolled iterations, incorporating data consistency and learned regularization terms. The initial 6 iterations focused on parallel imaging and the remaining 11 iterations focused on denoising. Then, the resulting images underwent further refinement using a deep learning-based super-resolution network with a pixel shuffle architecture [20] to enhance spatial resolution. The training of the k-space to image DL reconstruction included 500,000 single-shot DWI images of different body regions collected from volunteers scanned on different clinical 1.5 T and 3 T systems (MAGNETOM, Siemens Healthineers, Forchheim, Germany). The training pairs were generated by retrospectively doubling the acceleration factor. The training of the image-based super resolution network was performed on low-resolution images generated from the previously mentioned dataset by down sampling the spatial resolution by factor two. Training was conducted offline using PyTorch on an NVIDIA Tesla V100-SXM2 (16 GB Memory) GPU cluster at Siemens Healthineers, and the frozen networks were embedded directly into the scanner’s C + + reconstruction pipeline for assessment. The DWI specific reconstruction steps such as averaging and ADC calculation were done in the same fashion of standard DWI reconstruction.

### Image analysis

Image quality was assessed using commercial software (EV Insite R, PSP Corporation, Tokyo, Japan).

For the qualitative evaluation, two radiologists with nine and 25 years of experience independently evaluated the visualization of the pancreatic shape, main pancreatic duct (MPD), and the conspicuity of solid lesions on diffusion-weighted images (b = 1000 s/mm2) across all sequences. A 5-point scale was used for each category: for visualization of pancreatic shape and conspicuity of solid lesions, scores 1–5 signified not visible, poor, visible, clear, excellent clear (equal to fat-suppressed T1-weighted image regarding pancreatic shape), respectively; visualization of MPD: scores 1–5 signified not identifiable in the whole pancreas, identifiable in part of the pancreas, identifiable in about half pancreas, identifiable in more than half of the pancreas, and identifiable in the whole pancreas, respectively.

For quantitative analysis, the two radiologists manually set regions of interest (ROIs) by consensus in the pancreatic parenchyma, solid lesions (if present), and left erector spinae muscle. ROIs were as large as possible, ideally > 50 mm^2^. These ROIs were placed in the same axial section. In cases with solid lesions, ROIs were placed first on the most significant section of the lesion, followed by corresponding ROIs on the pancreatic parenchyma and erector spinae muscle in the same section, if possible. If the ROI could not be placed in the pancreatic parenchyma due to atrophy or other factors, the ROIs were placed in a different section that met the size criteria. Pancreas SNR and pancreas-to-muscle signal intensity ratio (PM-SIR) for evaluating pancreas conspicuity, lesion-to-pancreas signal-intensity ratio (LP-SIR) for evaluating conspicuity of pancreatic solid lesion were calculated as follows:

$${\text{SNR }} = {\text{ SI}}_{{{\text{pancreas}}}} /{\text{SD}}_{{{\text{pancreas}}}}$$

$${\text{PM}} - {\text{SIR }} = {\text{ SI}}_{{{\text{pancreas}}}} /{\text{SI}}_{{{\text{muscle}}}}$$

$${\text{LP}} - {\text{SIR}} = {\text{ SI}}_{{{\text{lesion}}}} /{\text{SI}}_{{{\text{pancreas}}}}$$

(SI_pancreas_: average SI of the pancreas, SI_muscle_: average SI of the muscle, SI_lesion_: average SI of the pancreatic solid lesion, and SD_pancreas_: standard deviation of the SI of the pancreas.)

Lesion-to-pancreas contrast-to-noise ratio was not calculated due to the parallel imaging technique, which the noise.

### Statistical analysis

For the qualitative analysis, inter-rater agreement was assessed using weighted kappa statistic. Kappa values were interpreted as follows: ≤ 0.20, poor agreement; 0.21–0.40, fair agreement; 0.41–0.60, moderate agreement; 0.61–0.80, good agreement; and 0.81–1.00, excellent agreement.

Statistical analyses were performed using SPSS version 29 (IBM, Armonk, NY, USA). The Friedman test with Bonferroni correction was applied to assess differences in pancreatic shape visualization, MPD, apparent diffusion coefficient (ADC) values of the pancreatic parenchyma and solid lesions, SNR, PM-SIR, and LP-SIR. A *P* value < 0.05 was considered statistically significant.

## Results

**Fig. 2:**
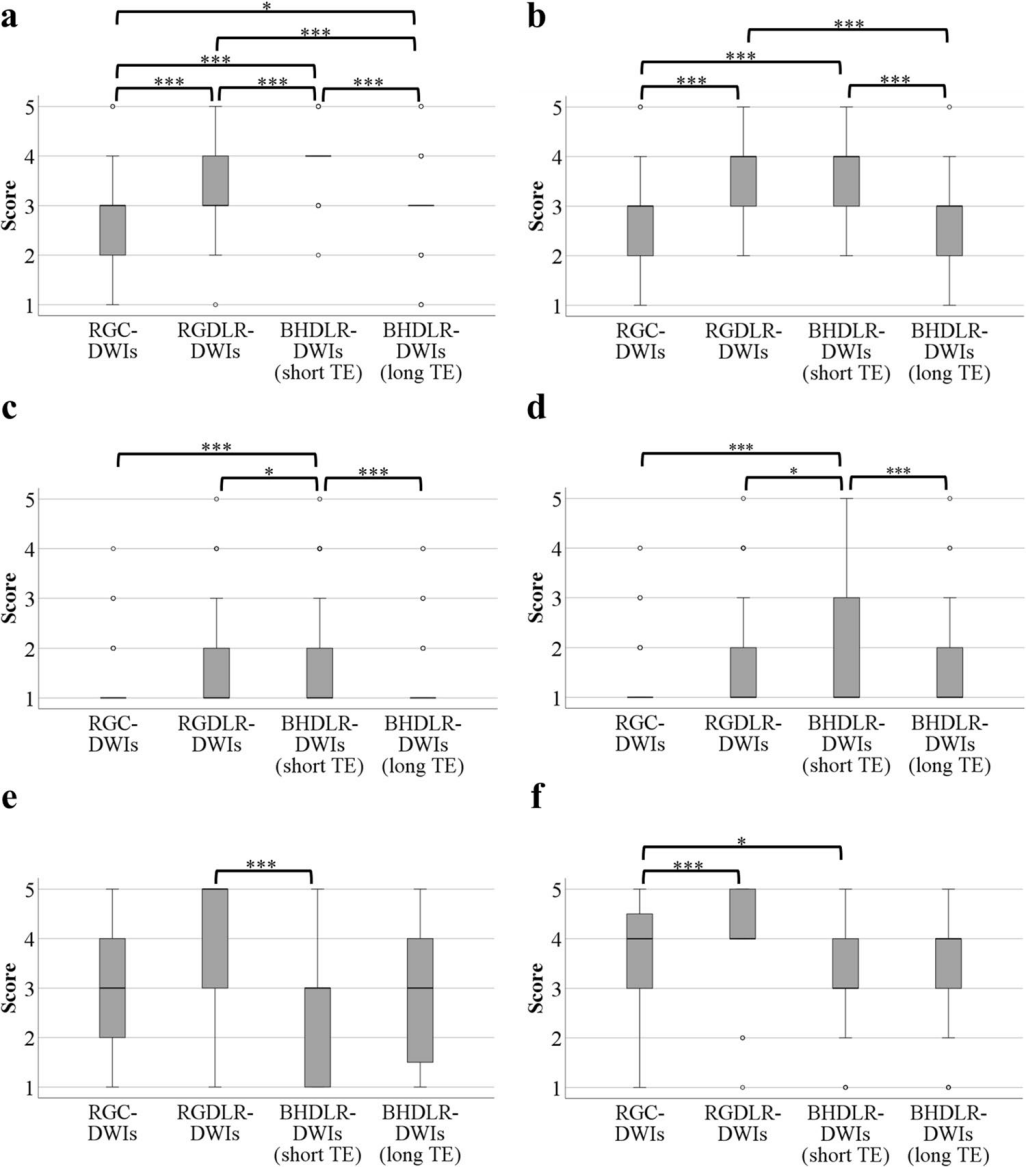
Qualitative evaluation. Two radiologists independently evaluated diffusion-weighted images (b = 1000 s/mm2) using a 5-point scale. **a** Pancreatic shape visualization by reader 1; **b** Pancreatic shape visualization by reader 2; **c** MPD visualization by reader 1; **d** MPD visualization by reader 2; **e** Solid lesions conspicuity by reader 1; and **f** Solid lesions conspicuity by reader 2. *P < 0.05; **P < 0.01; and ***P < 0.001. *RGC-DWIs* respiratory-gated conventional diffusion-weighted images, *RGDLR-DWIs* research respiratory-gated diffusion-weighted images with deep learning-based reconstruction, *BHDLR-DWIs* research breath-hold diffusion-weighted images with deep learning-based reconstruction, *MPD* main pancreatic duct

**Fig. 3:**
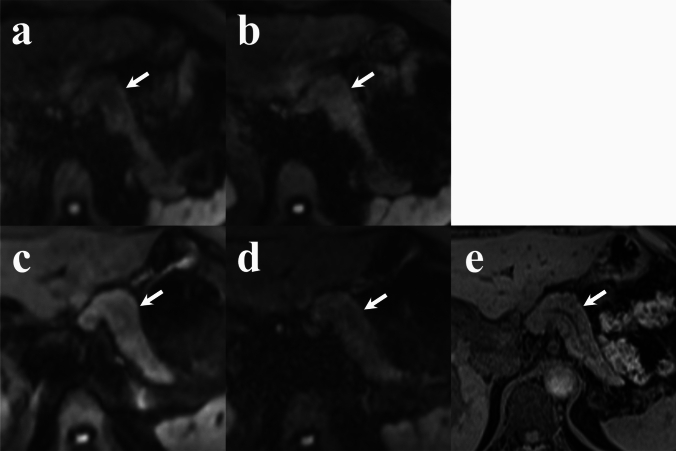
Comparison of pancreatic shape visualization (arrows) across diffusion-weighted images (b = 1000 s/mm^2^). **a** RGC-DWIs; **b** RGDLR-DWIs; **c** BHDLR-DWIs (short TE); **d** BHDLR-DWIs (long TE); and **e** Fat-suppressed T1-weighted image. BHDLR-DWIs (short TE) show the clearest pancreas morphology depiction. *RGC-DWIs* respiratory-gated conventional diffusion-weighted images, *RGDLR-DWIs* research respiratory-gated diffusion-weighted images with deep learning-based reconstruction, *BHDLR-DWIs* research breath-hold diffusion-weighted images with deep learning-based reconstruction

**Fig. 4:**
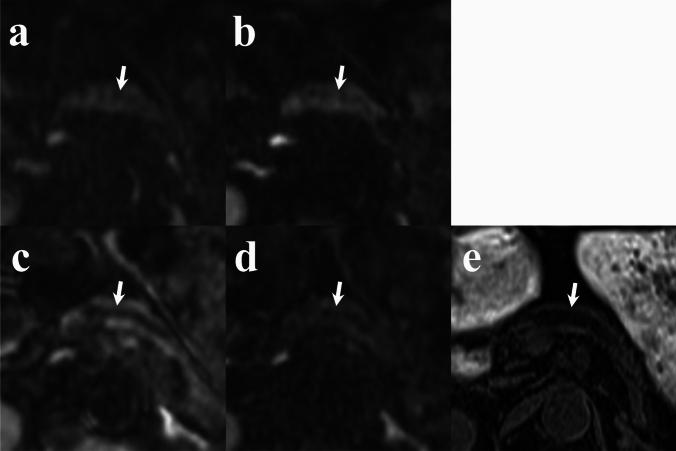
Comparison of MPD visualization (arrows) across diffusion-weighted images (b = 1000 s/mm^2^). **a** RGC-DWIs; **b** RGDLR-DWIs; **c** BHDLR-DWIs (short TE); **d** BHDLR-DWIs (long TE); and **e** Fat-suppressed T1-weighted image. BHDLR-DWIs (short TE) show the clearest MPD depiction. *RGC-DWIs* respiratory-gated conventional diffusion-weighted images, *RGDLR-DWIs* research respiratory-gated diffusion-weighted images with deep learning-based reconstruction, *BHDLR-DWIs* research breath-hold diffusion-weighted images with deep learning-based reconstruction, *MPD* main pancreatic duct

**Fig. 5:**
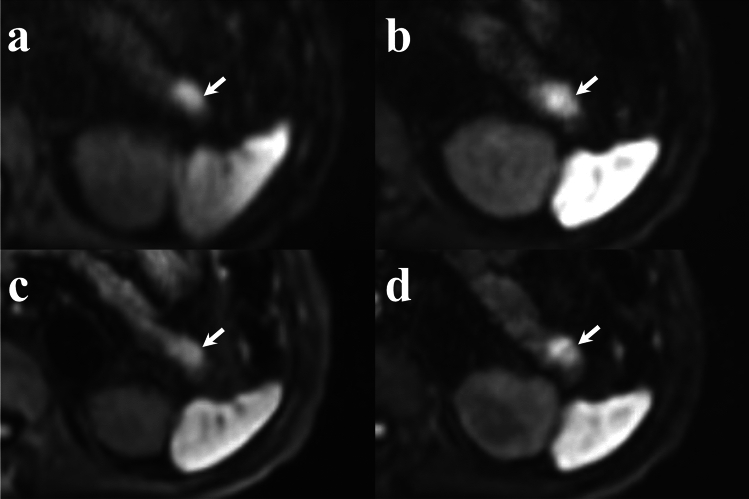
Comparison of pancreatic cancer visualization (arrows) on diffusion-weighted images (b = 1000 s/mm^2^). **a** RGC-DWIs; **b** RGDLR-DWIs; **c** BHDLR-DWIs (short TE); and **d** BHDLR-DWIs (long TE). The lesion shows the most distinct high-signal intensity on RGDLR-DWI and the least distinct signal intensity on BHDLR-DWI (short TE). *RGC-DWIs* respiratory-gated conventional diffusion-weighted images, *RGDLR-DWIs* research respiratory-gated diffusion-weighted images with deep learning-based reconstruction, *BHDLR-DWIs* research breath-hold diffusion-weighted images with deep learning-based reconstruction, *MPD* main pancreatic duct

**Fig. 6:**
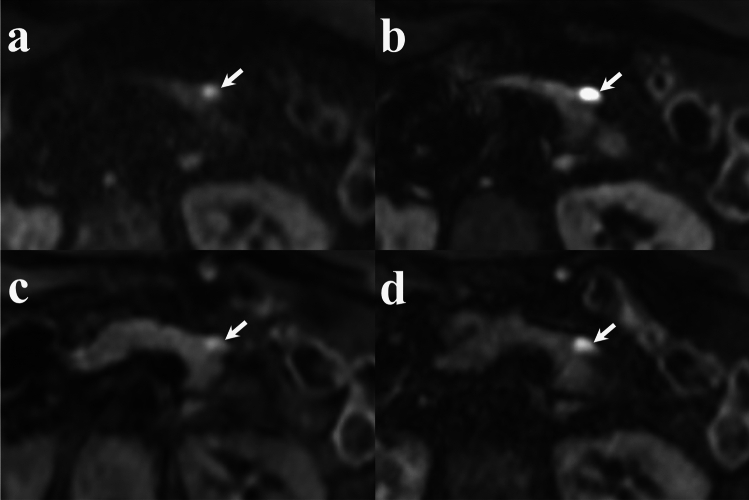
Comparison of neuroendocrine neoplasm visualization (arrows) on diffusion-weighted images (b = 1000 s/mm^2^). **a** RGC-DWIs; **b** RGDLR-DWIs; **c** BHDLR-DWIs (short TE); and **d** BHDLR-DWIs (long TE). The lesion shows the most distinct high-signal intensity on RGDLR-DWI and the least distinct signal intensity on BHDLR-DWI (short TE). *RGC-DWIs* respiratory-gated conventional diffusion-weighted images, *RGDLR-DWIs* research respiratory-gated diffusion-weighted images with deep learning-based reconstruction, *BHDLR-DWIs* research breath-hold diffusion-weighted images with deep learning-based reconstruction, *MPD* main pancreatic duct

**Fig. 7:**
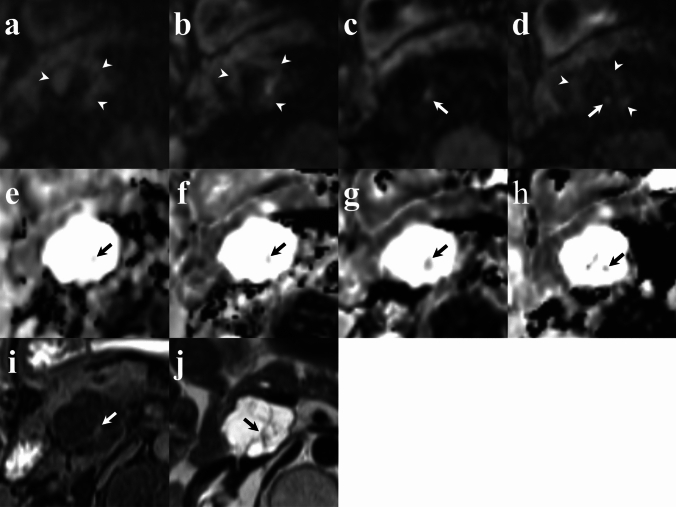
Comparison of intraductal papillary mucinous neoplasm with solid lesion (arrows) across sequences. **a** RGC-DWIs (b = 1000 s/mm^2^); **b** RGDLR-DWIs (b = 1000 s/mm^2^); **c** BHDLR-DWIs (short TE) (b = 1000 s/mm^2^); (d) BHDLR-DWIs (long TE) (b = 1000 s/mm^2^); **e** ADC map of RGC-DWIs; **f** ADC map of RGDLR-DWIs; **g** ADC map of BHDLR-DWIs (short TE); **h** ADC map of BHDLR-DWIs (short TE); (**i**) Suppressed T1-weighted image; and **j** HASTE. BHDLR-DWIs (short TE) showed a high signal only at the nodule, but in other sequences, high signal areas were scattered in the liquid part of the cyst, making it difficult to recognize the high signal of the nodule. BHDLR-DWIs (short TE) showed the clearest nodule depiction on the ADC map. *RGC-DWIs* respiratory-gated conventional diffusion-weighted images, *RGDLR-DWIs* research respiratory-gated diffusion-weighted images with deep learning-based reconstruction, *BHDLR-DWIs* research breath-hold diffusion-weighted images with deep learning-based reconstruction, *MPD* main pancreatic duct, *ADC* apparent diffusion coefficient

In the qualitative analysis, scores < 3 were considered non-diagnostic, whereas scores ≥ 3 were diagnostic. Kappa values were as follows: 0.551 (95% confidence interval [CI], 0.455–0.648) for pancreatic shape, 0.745 (95% CI, 0.652–0.838) for MPD, and 0.501 (95% CI, 0.341–0.661) for solid lesions, indicating moderate to good agreement between readers. The qualitative analysis results are shown in Fig. 2 and Table 3. Median scores for pancreatic shape in BHDLR-DWI (short TE) by Reader 1 were the highest and Reader 1 rated BHDLR-DWIs (short TE) significantly higher than all other sequences (*P* < 0.001). Those in BHDLR-DWI (short TE) by Reader 2 were similar to RGDLR-DWIs but higher than RGC-DWIs and BHDLR-DWIs (long TE). Reader 2 also rated BHDLR-DWIs (short TE) significantly higher than RGC-DWIs and BHDLR-DWIs (long TE) (*P* < 0.001), with no significant difference compared to RGDLR-DWIs. Figure 3 shows that BHDLR-DWIs (short TE) provided the clearest depiction of pancreatic morphology. Median score and Q1 values for MPD visualization by both readers were the same for all sequences. However, Q3 values in BHDLR-DWIs (short TE) by Reader 2 were the highest, and those by Reader 1 was the same as RGDLR-DWIs but higher than RGC-DWIs and BHDLR-DWIs (long TE). Both readers rated BHDLR-DWIs (short TE) significantly higher than other sequences (RGC-DWIs, *P* < 0.001; RGDLR-DWIs, *P* < 0.05; BHDLR-DWIs (long TE), *P* < 0.001). Figure 4 shows that MPD is most clearly recognized in BHDLR-DWIs (short TE). Median scores for solid lesions in BHDLR-DWIs (short TE) by Reader 1 were similar to RGC-DWIs and BHDLR-DWIs (long TE), but lower than RGDLR-DWIs. Those in BHDLR-DWIs (short TE) by Reader 2 was the lower than other sequences. Those in all sequences except BHDLR-DWIs (short TE) were similar, but Q1 and Q3 were the highest in RGDLR-DWIs by Reader 2. Reader 1 rated RGDLR-DWIs significantly higher than BHDLR-DWIs (short TE) (*P* < 0.001), but no significant difference was observed between RGDLR-DWIs and BHDLR-DWIs for Reader 2. Reader 2 rated RGDLR-DWIs significantly higher than RGC-DWIs (*P* < 0.001), but this was not observed for Reader 1. Figures 5 and 6 show neuroendocrine neoplasm and pancreatic cancer cases, respectively, where lesions had the highest signal intensity on RGDLR-DWIs and the weakest on BHDLR-DWI (short TE). In one case of IPMN with a solid lesion, BHDLR-DWIs (short TE) showed high signal only at the nodule, whereas other sequences showed scattered high signals in the liquid portion of the cyst, making it difficult to distinguish the nodule. On the ADC map, BHDLR-DWIs (short TE) most clearly visualized the nodule (Fig. 7).

**Table 3:** Evaluation score in qualitative analysis

	RGC-DWIs	RGDLR-DWIs	BHDLR-DWIs (short TE)	BHDLR-DWIs (long TE)
Pancreatic shape	R1 3(2,3)	R1 3(3,4)	R1 4(4,4)	R1 3(3,3)
	R2 3(2,3)	R2 4(3,4)	R2 4(3,4)	R2 3(2,3)
Main pancreatic duct	R1 1(1,1)	R1 1(1,2)	R1 1(1,2)	R1 1(1,1)
	R2 1(1,1)	R2 1(1,2)	R2 1(1,3)	R2 1(1,2)
Solid lesion	R1 3(2,4)	R1 5(3,5)	R1 3(1,3)	R1 3(1.5,4)
	R2 4(3,4.5)	R2 4(4,5)	R2 3(3,4)	R2 4(3,4)

Variables are expressed as medians; numbers in parentheses are Q1,Q3; *RGC-DWIs* respiratory-gated conventional diffusion-weighted images, *RGDLR-DWIs*\, research respiratory-gated diffusion-weighted images with deep learning reconstruction, *BHDLR-DWIs* research breath-hold diffusion-weighted images with deep learning reconstruction, *R1* reader 1, *R2* reader 2

**Fig. 8:**
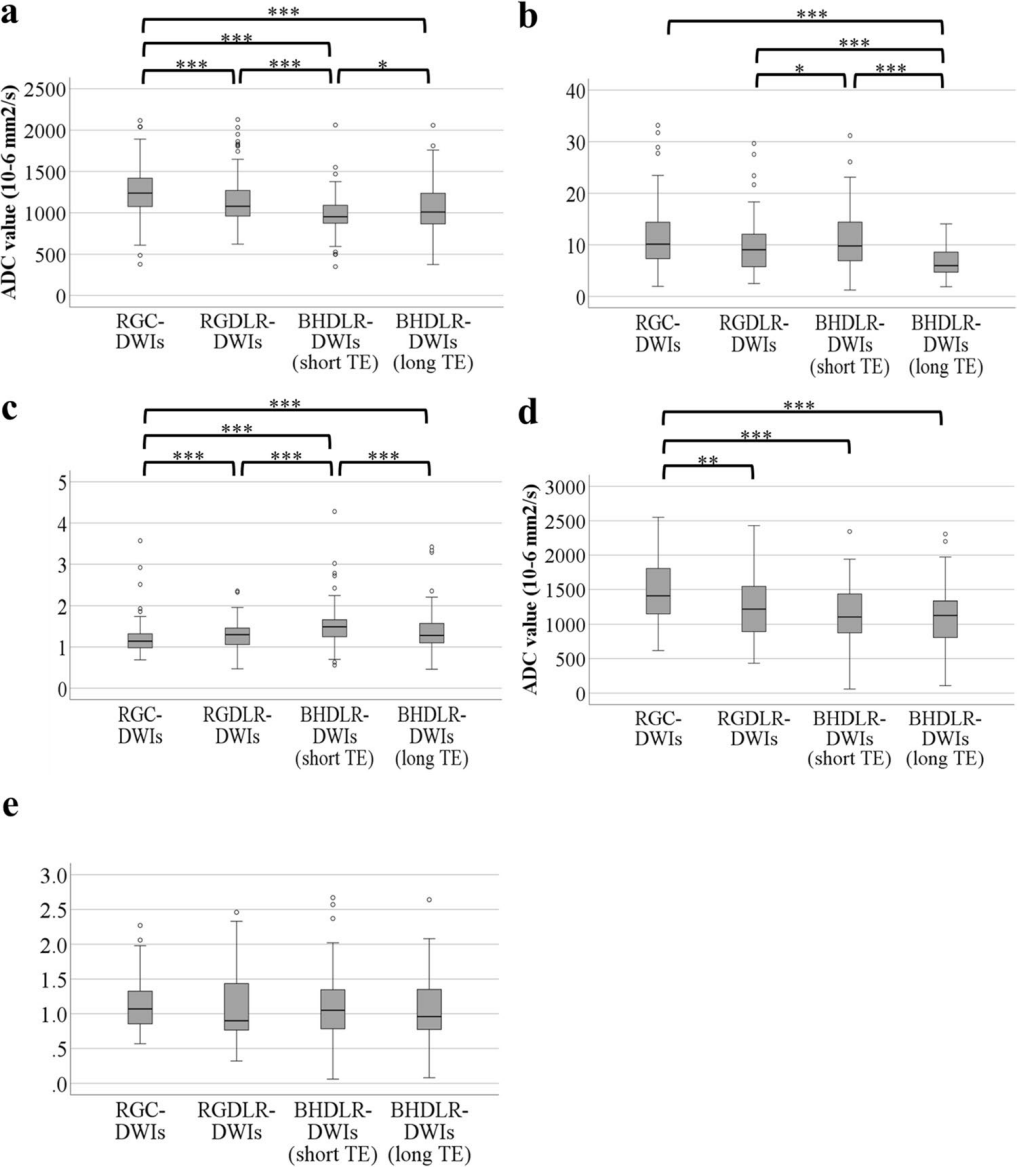
Quantitative evaluation. **a** ADC values of pancreatic parenchyma; **b** SNR; **c** PM-SIR; **d** ADC values of solid lesions; and **e** LP-SIR. *P < 0.05; **P < 0.01; and ***P < 0.001. RGC-DWIs: respiratory-gated conventional diffusion-weighted images; *RGDLR-DWIs* research respiratory-gated diffusion-weighted images with deep learning-based reconstruction, *BHDLR-DWIs* research breath-hold diffusion-weighted images with deep learning-based reconstruction, *MPD* main pancreatic duct, *ADC* apparent diffusion coefficient, *SNR* signal-to-noise ratio, *PM-SIR* pancreas-to-muscle signal-intensity ratio, *LP-SIR* lesion-to-pancreas signal-intensity ratio

ROIs were placed in the pancreatic parenchyma of the head, body and tail in 5, 10 and 102 patients respectively throughout the entire study. The median ROI sizes for quantitative analysis were 53.67 (10.25–272.27), 70.99 (35.03–316.19), and 31.3 (10.25–880.99) mm^2^ for pancreatic parenchyma, left erector spinae muscle and solid lesions, respectively. Quantitative analysis results are shown in Fig. 8. The ADC values of the pancreatic parenchyma were significantly lower in BHDLR-DWIs (short TE) compared to other sequences (RGC-DWIs, *P* < 0.001; RGDLR-DWIs, *P* < 0.01; BHDLR-DWIs (long TE), *P* < 0.05). Furthermore, the ADC values of RGDLR-DWIs were significantly lower compared to RGC-DWIs (*P* < 0.001). SNR was highest in RGC-DWIs but not significantly different from RGDLR-DWIs and BHDLR-DWIs (short TE). PM-SIR was significantly higher in BHDLR-DWIs (short TE) compared to other sequences (*P* < 0.001). The ADC values of solid lesions were lowest in BHDLR-DWIs (short TE), but no significant difference was found between RGDLR-DWIs and BHDLR-DWIs (short TE). The ADC values of solid lesions in RGDLR-DWIs were significantly lower compared to RGC-DWIs (*P* < 0.01). Although LP-SIR in RGDLR-DWIs were the lowest, the difference was not significant.

## Discussion

BHDLR-DWI (short TE) performed best for morphologic delineation of the pancreas as shown by qualitative analysis and PM-SIR and had the lowest ADC values. It was also superior in MPD depiction, likely due to the clear delineation of the pancreatic parenchyma, enhancing the contrast between the low-signal main pancreatic duct and the pancreatic parenchyma signal in BHDLR-DWIs (short TE). However, in solid lesion assessment, BHDLR-DWIs (short TE) ranked lowest in qualitative evaluation, possibly because the clearer delineation of the pancreatic parenchyma reduced the contrast between the high-signal solid lesions and the parenchyma. Despite its limitations in solid lesion depiction, BHDLR-DWIs (short TE) was the only sequence to show high signal intensity at the solid lesion of IPMN without interference from the liquid part of the cyst, likely due to the short TE, which reduces T2 shine-through effects. Notably, this characteristic of BHDLR-DWIs (short TE) might be valuable in identifying solid lesions that contribute to assessing malignancy in IPMN, though further study is needed. The lower ADC values observed in the pancreatic parenchyma and solid lesions on BHDLR-DWIs (short TE) compared to BHDLR-DWIs (long TE) align with existing literature showing lower ADC values with shorter TE [21].

When comparing RGC-DWIs and RGDLR-DWIs to assess DLR impact, RGDLR-DWIs showed significantly lower ADC values compared to RGC-DWIs for pancreatic parenchyma and solid lesions. Quantitative analysis also showed that RGDLR-DWIs were higher than RGC-DWIs across all metrics, particularly for solid lesions, which was the highest in all sequences. Takayama et al. reported an improvement in pancreatic contour sharpness with DLR, although without statistical significance [16]. In this study, RGDLR-DWIs yielded higher pancreatic shape scores than RGC-DWIs for both readers, with one reader showing a significant difference, consistent with previous findings. Takayama et al. also observed that DLR altered ADC values in the pancreas and pancreatic cysts [16], which aligns with our results. As far as we know, there is no report on DLR-related ADC changes for pancreatic solid lesions, but Bae et al. found significant ADC reductions with DLR in hepatic malignancies [22], indicating that such changes in pancreatic solid lesions are plausible. It is thought that the reduction in noise and artifacts through DLR contributes to these changes in ADC values [22–24]. Finally, one difference between RGC-DWIs and RGDLR-DWIs is the acquisition time, which can be reduced with DLR, providing an additional clinical benefit.

Regarding SNR, although RGC-DWIs had the highest value, the difference was not significant when compared to RGDLR-DWIs and BHDLR-DWIs (short TE), indicating that RGDLR-DWIs and BHDLR-DWIs (short TE) offer clinically acceptable SNRs.

Although LP-SIR in RGDLR-DWIs were the lowest, the difference was not significant. This result was contrary to qualitative analysis and might be due to the possibility that small lesions were not accurately measured and the fact that the contrast between the pancreatic parenchyma and the solid lesion was not important when evaluating solid lesions of IPMN.

This study has some limitations. First, it was conducted at a single institution, with a relatively small sample size for solid lesions. Second, ROI was based on 2D evaluation, not 3D. Furthermore, measuring small solid lesions may not have been entirely accurate. Third, multiple changes in MRI parameters across four sequences make it difficult to determine isolated impact of each modification. DWI with breath-hold might have modifications such as lower SNR and higher ADC values due to shorter TR compared to that with respiratory-gating, and lower ADC values due to the use of 3D-Diagonal instead of 3-scan trace [25]. Finally, pathologic diagnoses were unavailable for all patients.

In conclusion, BHDLR-DWIs (short TE) provided the best image quality for pancreatic morphology, whereas RGDLR-DWIs demonstrated superior image quality for evaluating pancreatic solid lesions.

## References

[1] Bammer R. Basic principles of diffusion-weighted imaging. Eur J Radiol. 2003;45:169–84. 10.1016/s0720-048x(02)00303-0. 12595101 10.1016/s0720-048x(02)00303-0

[2] Barral M, Taouli B, Guiu B, Koh DM, Luciani A, Manfredi R, et al. Diffusion-weighted MR imaging of the pancreas: current status and recommendations. Radiology. 2015;274:45–63. 10.1148/radiol.14130778. 25531479 10.1148/radiol.14130778

[3] Tang MY, Zhang XM, Chen TW, Huang XH. Various diffusion magnetic resonance imaging techniques for pancreatic cancer. World J Radiol. 2015;7:424–37. 10.4329/wjr.v7.i12.424. 26753059 10.4329/wjr.v7.i12.424 PMC4697117

[4] Harder FN, Jung E, Weiss K, Graf MM, Kamal O, McTavish S, et al. Computed high-b-value high-resolution DWI improves solid lesion detection in IPMN of the pancreas. Eur Radiol. 2023;33:6892–901. 10.1007/s00330-023-09661-6. 37133518 10.1007/s00330-023-09661-6 PMC10511579

[5] Fattahi R, Balci NC, Perman WH, Hsueh EC, Alkaade S, Havlioglu N, et al. Pancreatic diffusion-weighted imaging (DWI): comparison between mass-forming focal pancreatitis (FP), pancreatic cancer (PC), and normal pancreas. J Magn Reson Imaging. 2009;29:350–6. 10.1002/jmri.21651. 19161187 10.1002/jmri.21651

[6] Bartoli M, Barat M, Dohan A, Gaujoux S, Coriat R, Hoeffel C, Soyer P, et al. CT and MRI of pancreatic tumors: an update in the era of radiomics. Jpn J Radiol. 2020;38:1111–24. 10.1007/s11604-020-01057-6. 33085029 10.1007/s11604-020-01057-6

[7] Park MJ, Kim YK, Choi SY, Rhim H, Lee WJ, Choi D. Preoperative detection of small pancreatic carcinoma: value of adding diffusion-weighted imaging to conventional MR imaging for improving confidence level. Radiology. 2014;273:433–43. 10.1148/radiol.14132563. 24991989 10.1148/radiol.14132563

[8] Kwee TC, Takahara T, Ochiai R, Nievelstein RAJ, Luijten PR. Diffusion-weighted whole-body imaging with background body signal suppression (DWIBS): features and potential applications in oncology. Eur Radiol. 2008;18:1937–52. 10.1007/s00330-008-0968-z. 18446344 10.1007/s00330-008-0968-z PMC2516183

[9] Dietrich O, Biffar A, Baur-Melnyk A, Reiser MF. Technical aspects of MR diffusion imaging of the body. Eur J Radiol. 2010;76:314–22. 10.1016/j.ejrad.2010.02.018. 20299172 10.1016/j.ejrad.2010.02.018

[10] Kartalis N, Loizou L, Edsborg N, Segersvärd R, Albiin N. Optimising diffusion-weighted MR imaging for demonstrating pancreatic cancer: a comparison of respiratory-triggered, free-breathing and breath-hold techniques. Eur Radiol. 2012;22:2186–92. 10.1007/s00330-012-2469-3. 22549106 10.1007/s00330-012-2469-3

[11] Higaki T, Nakamura Y, Tatsugami F, Nakaura T, Awai K. Improvement of image quality at CT and MRI using deep learning. Jpn J Radiol. 2019;37:73–80. 10.1007/s11604-018-0796-2. 30498876 10.1007/s11604-018-0796-2

[12] Afat S, Herrmann J, Almansour H, Benkert T, Weiland E, Hölldobler T, et al. Acquisition time reduction of diffusion-weighted liver imaging using deep learning image reconstruction. Diagn Interv Imaging. 2023;104:178–84. 10.1016/j.diii.2022.11.002. 36787419 10.1016/j.diii.2022.11.002

[13] Tanabe M, Higashi M, Yonezawa T, Yamaguchi T, Iida E, Furukawa M, et al. Feasibility of high-resolution magnetic resonance imaging of the liver using deep learning reconstruction based on the deep learning denoising technique. Magn Reson Imaging. 2021;80:121–6. 10.1016/j.mri.2021.05.001. 33971240 10.1016/j.mri.2021.05.001

[14] Ichinohe F, Oyama K, Yamada A, Hayashihara H, Adachi Y, Kitoh Y, et al. Usefulness of breath-hold fat-suppressed T2-weighted images with deep learning-based reconstruction of the liver: comparison to conventional free-breathing turbo spin echo. Invest Radiol. 2023;58:373–9. 10.1097/RLI.0000000000000943. 36728880 10.1097/RLI.0000000000000943

[15] Mulé S, Kharrat R, Zerbib P, Massire A, Nickel MD, Ambarki K, et al. Fast T2-weighted liver MRI: image quality and solid focal lesions conspicuity using a deep learning accelerated single breath-hold HASTE fat-suppressed sequence. Diagn Interv Imaging. 2022;103:479–85. 10.1016/j.diii.2022.05.001. 35597761 10.1016/j.diii.2022.05.001

[16] Takayama Y, Sato K, Tanaka S, Murayama R, Goto N, Yoshimitsu K. Deep learning-based magnetic resonance imaging reconstruction for improving the image quality of reduced-field-of-view diffusion-weighted imaging of the pancreas. World J Radiol. 2023;15:338–49. 10.4329/wjr.v15.i12.338. 38179202 10.4329/wjr.v15.i12.338 PMC10762521

[17] Altmann S, Grauhan NF, Mercado MAA, Steinmetz S, Kronfeld A, Paul R, et al. Deep learning accelerated brain diffusion-weighted MRI with super resolution processing. Acad Radiol. 2024;31:4171–82. 10.1016/j.acra.2024.02.049. 38521612 10.1016/j.acra.2024.02.049

[18] Jeong J, Yeom SK, Choi IY, Cha SH, Han JS, Lee CH, et al. Deep learning image reconstruction of diffusion-weighted imaging in evaluation of prostate cancer focusing on its clinical implications. Quant Imaging Med Surg. 2024;14:3432–46. 10.21037/qims-23-1379 10.21037/qims-23-1379 PMC11074768 38720859

[19] Hammernik K, Klatzer T, Kobler E, Recht MP, Sodickson DK, Pock T, et al. Learning a variational network for reconstruction of accelerated MRI data. Magn Reson Med. 2018;79:3055–71. 10.1002/mrm.26977. 29115689 10.1002/mrm.26977 PMC5902683

[20] Shi W, Caballero J, Huszar F, Totz J, Aitken AP, Bishop R, et al. Real-time single image and video super-resolution using an efficient sub-pixel convolutional neural network. In: Proceedings of the IEEE conference on computer vision and pattern recognition. New York: IEEE; 2016 p. 1874–83. 10.1109/CVPR.2016.207.

[21] Celik A. Effect of imaging parameters on the accuracy of apparent diffusion coefficient and optimization strategies. Diagn Interv Radiol. 2016;22:101–7. 10.5152/dir.2015.14440. 26573977 10.5152/dir.2015.14440 PMC4712890

[22] Bae SH, Hwang J, Hong SS, Lee EJ, Jeong J, Benkert T, et al. Clinical feasibility of accelerated diffusion weighted imaging of the abdomen with deep learning reconstruction: comparison with conventional diffusion weighted imaging. Eur J Radiol. 2022;154: 110428. 10.1016/j.ejrad.2022.110428. 35797791 10.1016/j.ejrad.2022.110428

[23] Iima M, Partridge SC, Le Bihan D. Six DWI questions you always wanted to know but were afraid to ask: clinical relevance for breast diffusion MRI. Eur Radiol. 2020;30:2561–70. 10.1007/s00330-019-06648-0. 31965256 10.1007/s00330-019-06648-0

[24] Chen Q, Fang S, Yuchen Y, Li R, Deng R, Chen Y, et al. Clinical feasibility of deep learning reconstruction in liver diffusion-weighted imaging: improvement of image quality and impact on apparent diffusion coefficient value. Eur J Radiol. 2023;168: 111149. 10.1016/j.ejrad.2023.111149. 37862927 10.1016/j.ejrad.2023.111149

[25] Hectors SJ, Wagner M, Corcuera-Solano I, Kang M, Stemmer A, Boss MA, et al. C comparison between 3-scan trace and diagonal body diffusion-weighted imaging acquisitions: a phantom and volunteer study. Tomography. 2016;2:411–420. 10.18383/j.tom.2016.00229 10.18383/j.tom.2016.00229 PMC5416814 28480331

